# Arsenic Poisoning

**Published:** 1891-04

**Authors:** 


### ARSENIC POISONING.

(From the *Boston Daily Traveler*.)

The regular monthly meeting of the Boston Homœopathic Medical Society was held Thursday evening, February 5th, at No. 98 Boylston Street, and was called to order at 7.40 P. M., by the President, Dr. G. R. Southwick, who occupied the chair.

A considerable amount of routine business was transacted, after which the Society proceeded to the discussion of the dangers of poisoning from arsenic used as coloring matter.

Prof. E. E. Calder, of Brown University, exhibited several simple tests for the detection of arsenic in wall-papers. He said:

The true arsenic, as it is commonly termed, applies to that compound of the element more commonly designated as white, or chemically arsenious oxide.

The most common application of arsenic is its use in the manufacture of pigments. The application of white arsenic in this direction may be considered as two-fold in its character, as in the manufacture of coloring matter. There is a class of coloring substances in which arsenic enters as an essential constituent, in fact, imparting to the color its brilliancy and value. A very large class of colors is in constant use in the preparation of which the arsenic plays simply a minor part, used as an oxidizing agent, and existing in the finished color only in very minute but appreciable traces.

The speaker told where and from what metals arsenic was most commonly obtained. Imperial green contains an arsenite of copper prepared by mixing a solution of acetate of copper with a solution of white arsenic. Schweinfurth's green is a mixture of arsenite and the acetate of copper. These two closely allied and similar compounds, from their comparative cheapness in connection with their exceedingly brilliant and rich green color, are very commonly and extensively used as green paints. These compounds also find common application in the staining of paper, for coloring light cotton fabrics, for preparation of artificial flowers, in manufacture of candies, etc.

Rouge yellow, an artist's color, contains ninety-seven per cent. of white arsenic, and is therefore extremely poisonous. Its application as a more common pigment is now superseded by the cheaper and comparatively innocuous chrome-yellow or chromate of lead pigment.

To the class of coloring matters not containing arsenic as an essential constituent, belong the aniline colors. The aniline red always contains some arsenic. When this color is used for tinting confectionery it is understood that the manufacturer will use some oxidizing agent other than white arsenic. It is thus clearly acknowledged that such a use of arsenic is not only not advisable but unnecessary. The aniline colors are employed in the industrial arts for numerous other purposes besides their great use as dyeing materials, as in the tinting of paper pulp, the staining of wall-papers, the preparations of water-colors, the manufacture of colored inks, the coloring of cosmetics, fancy soaps, perfumery, confectionery, etc.

By far the most prolific source of arsenical poisoning arises from the use of wall-papers, draperies, curtains, carpets, etc., containing arsenic. There is no question but that many of the materials used to adorn our homes to-day contain arsenic in greater or lesser quantities. A wall-paper can be made to contain no arsenic, because we often find samples on sale entirely free. If paper can be prepared without any additional cost, the public has a right to insist upon receiving the benefit, and the slightest trace of arsenic in a paper ought to condemn it for use. Regarding the prevalence of arsenic in fabrics the same may be said.

Dr. Talbot spoke upon the same subject, and, among other things, said:

The peculiar character of arsenic and its wonderful power of combination with other substances to produce a great variety of brilliant and enduring colors has brought it into a very extensive use, which has steadily and rapidly increased until it now enters into the manufacture of a very large variety of domestic articles, many of which are worn as clothing or brought into close contact with individuals, and there is hardly a household in the country but has more or less of this poison in some form within it. Aside from the large quantities produced from some of the mines in the West and from various other sources, the importations of arsenic into this country the last year amounted to about ten million pounds, thus furnishing more than two and one-half ounces to every man, woman, and child in the country.

Now, when we consider that two grains taken at once into the stomach is sufficient to cause death, the amount of this death-dealing substance which is imported is truly appalling. Fortunately the system can resist the poisonous effects of many substances, and especially of metallic substances, when introduced into it in small quantities and slowly, yet there are many instances in which persons have been directly and fatally poisoned, and a very much larger number of persons who have been seriously injured by contact and absorption of this poison. We have heard this evening of various articles of domestic use in which arsenic is incorporated. We sleep in bed-rooms the walls of which are hung with paper filled with arsenic. Our most beautiful draperies are equally loaded with this poison.

We sit upon sofas that every time they are compressed throw into the atmosphere this same poison. We wear clothing containing enough arsenic, if taken into the stomach, to produce a speedy death. Our little children are wrapped in beautiful shawls containing this same death-dealing drug. Their playthings are rendered more beautiful and attractive by this very poison. The papers in which their bon-bons and candies are enveloped are colored with arsenical preparations, and even the utensils in which our food is cooked are sometimes lined with this poison. Now, if any considerable proportion of arsenic is taken into the stomach at once, its effects are so uniformly severe that suspicion of poisoning is immediately aroused and search is made for the cause of it. But when it is taken very slowly the symptoms are so masked by many surrounding circumstances and conditions that even the most experienced physicians do not discover the cause.

The soreness of the throat, the difficulty of breathing, the nausea and vomiting, the pallor and weakness often are attributed to entirely different causes, and it may be months, or even years before the true cause is discovered. To-day one of the most honored citizens of Boston is lying on his death-bed, after two or more years of prostration and suffering, and it is only within the last few months that it was discovered that his urine was loaded with arsenic, which his system had been gradually absorbing from long-continued exposure to it. The nicer chemical tests of late years are discovering the same condition in many chronic invalids, while every physician has had cases, which, resisting all treatment, he has been obliged to send away from home into different surroundings before they could be relieved.

Arsenic taken into the system in this insidious manner not only produces the symptoms peculiar to itself, but from its depressing influence upon all the vital functions, renders it more susceptible to every form of disease to which it may be exposed. When we consider how our little children are often, from their earliest infancy, surrounded by this poison, and their systems thus rendered susceptible to other diseases, is it strange that the mortality among them is so great? At the very least, is it not our duty as physicians, knowing the great dangers which accompany this poison, to take every means in our power to protect our patients and the community from its influence? If a mad dog let loose in the community destroys but a single life, the public are aroused to the greatest excitement over it, and pass stringent laws to protect them from this danger.

Ought we not then to have laws which will protect us from the danger much greater and more insidious, and which is concealed under forms most attractive and allurring? * * *

The following resolutions were then passed.

WHEREAS, It is well known that arsenic is a virulent poison, of which two grains will produce a fatal result, and a much smaller quantity will cause serious injury to health.

That for the protection and safety of its people this State has passed laws directing every apothecary who sells even the smallest quantity of arsenic to label it "Poison," and imposes a heavy fine for neglect of such duty.

That this substance is used in large quantities in the manufacture of goods for domestic use, such as paper hangings, draperies, wearing apparel, children's toys, etc.

That many persons are poisoned through ignorantly using such articles, and often suffer loss of health and even life hereby.

*Therefore Resolved*, That in the opinion of this Society this State should pass such laws as will properly restrict the manufacture and sale of all articles for domestic use containing arsenic, by providing, among other things, that when articles containing such matter are offered for sale they shall be clearly and legibly marked to show that they contain poison, and by providing also that the violation of such laws shall be punished by fine or imprisonment or both.

*Resolved*, That a committee of five be appointed by this Society to co-operate with, and aid the committee of Massachusetts Homœopathic Medical Society in their efforts to secure proper restrictive legislation on this subject.

*Resolved*, That we call upon the other medical societies of the State, upon all the physicians, chemists, and scientists, as well as the citizens at large to aid us in this effort to protect the public health.

